# Efficacy analysis of enhanced recovery after surgery in laparoscopic-assisted radical resection of type I choledochal cyst

**DOI:** 10.3389/fped.2023.1191065

**Published:** 2023-06-21

**Authors:** Bing Zhang, Yifan Fang, Dianming Wu, Siqi Xie, Xuejuan Fang

**Affiliations:** Department of Pediatric Surgery, Fujian Children’s Hospital (Fujian Branch of Shanghai Children’s Medical Center), College of Clinical Medicine for Obstetrics & Gynecology and Pediatrics, Fujian Medical University, Fuzhou, China

**Keywords:** enhanced recovery after surgery, laparoscopy, choledochal cyst, children, pediatric surgery

## Abstract

**Objective:**

The objective of this study was to investigate the feasibility and effectiveness of laparoscopic-assisted radical resection of type I choledochal cyst (CC) guided by the principles of enhanced recovery after surgery (ERAS).

**Methods:**

A retrospective cohort study of type I CC admitted to our hospital between May 2020 and December 2021 were analyzed, a total of 41 patients with choledochal cyst underwent surgery during this period and 30 cases were selected based on inclusion and exclusion criteria. Patients (*n* = 15) who received the traditional treatment from May 2020 to March 2021 were included in the traditional group. Patients (*n* = 15) who received ERAS from April 2021 to December 2021 were included in the ERAS group. Both groups underwent surgery performed by the same surgical team. Preoperative data of the two groups were recorded, and relevant data were statistically analyzed and compared.

**Results:**

There was a statistically significant difference in the dose of opioids. Significant differences were observed between the ERAS and traditional groups in the results of the FLACC pain assessment scale on the 1st and 2nd day after surgery, time of gastric tube, urinary catheter and abdominal drainage tube removal, time of first defecation after operation, time of first eating after operation, time to reach full food intake, results of CRP, ALB, and ALT on the 3rd and 7th postoperative day, postoperative hospital stay, and total treatment cost. No significant differences were observed between the two groups in terms of gender, age, body weight, cyst size, preoperative CRP, ALB, ALT, intraoperative blood loss, operation time, and the number of cases converted to laparotomy. Neither the FLACC pain assessment scale on the 3rd day after surgery, the incidence of postoperative complications, nor the rate of readmission within 30 days showed significant differences.

**Conclusions:**

Laparoscopic-assisted radical resection of type I CC guided by the principles of ERAS is safe and effective for children. The ERAS concept demonstrated advantages over traditional laparoscopic surgery, including reduced opioid use, shorter time to first postoperative defecation, earlier resumption of postoperative feeding, shorter time to reach full feeding, shorter postoperative hospital stay, and lower total treatment cost.

## 1. Introduction

Choledochal cyst (CC), also known as congenital bile duct dilatation, is a disease that causes fever, abdominal pain, jaundice, and other symptoms in patients due to intrahepatic or extrahepatic bile duct dilatation. It is prevalent in Asian countries, with Japan having an incidence as high as 1/1,000 and a male to female ratio of about 1:3 (1). Complete resection of the lesion and Roux-en-Y anastomosis of hepatic duct and jejunum are the standard surgical methods for this disease. Enhanced Recovery After Surgery (ERAS) was first proposed by Danish surgeon Kehlet in the late 20th century (2). It refers to the use of evidence-based medicine by a multidisciplinary team to optimize preoperative, intraoperative, and postoperative procedures, reduce the physical and psychological trauma of patients, shorten the length of hospital stay, and promote the rapid recovery of patients. While ERAS has been successfully developed in the field of adult surgery and has established guidelines or consensus for a variety of diseases, it is a late starter in pediatric surgery. The operative of CC is a typical four-stage operation in pediatric surgery, the children will face more complex stress response than adults during perioperative period, so ERAS is more urgent to be needed.

The purpose of this study is to apply the ERAS concept to the perioperative process of CC and objectively evaluate its safety, limitations, and clinical application value.

## 2. Materials and methods

### 2.1. General information

A retrospective cohort study of type I CC admitted to our hospital between May 2020 and December 2021 were analyzed, a total of 41 patients with choledochal cyst underwent surgery during this period and 30 cases were selected based on inclusion and exclusion criteria. Patients (*n* = 15) who received the traditional treatment from May 2020 to March 2021 were included in the traditional group. Patients (*n* = 15) who received ERAS from April 2021 to December 2021 were included in the ERAS group.

The inclusion criteria were: (1) confirmation of type I CC via color ultrasound and magnetic resonance cholangiopancreatography (MRCP), (2) age between 3 months and 18 years old, (3) agreement from family members to undergo laparoscopic surgery, and (4) operation performed by the same surgical and anesthesia teams.

The exclusion criteria were: (1) preoperative peritonitis, (2) previous cholecystostomy or choledochostomy due to severe biliary tract infection, (3) abnormal coagulation function that could not be corrected prior to surgery, (4) severe liver function damage, and (5) patients with severe postoperative complications who should not continue with the ERAS program.

This study was approved by the Medical Ethics Committee of Fujian Children's Hospital (2022ETKLR08045), and informed consent was obtained from the family members.

### 2.2. Treatment methods

#### 2.2.1. Preoperative examination of all hospitalized children

All hospitalized children underwent preoperative examinations including blood routine, C-reactive protein (CRP), urine routine, fecal routine, biochemical examination, hematural amylase, coagulation function, chest x-ray, electrocardiogram, color ultrasound, and MRCP.

#### 2.2.2. Perioperative management

Table 1 shows the preoperative, intraoperative, and postoperative management for both ERAS and traditional groups, respectively.

**Table 1:** Comparison of preoperative management measures between ERAS group and traditional group.

Group	ERAS group	Traditional group
Preoperative education and psychological counseling	Through pictures, videos and other means of education, and give psychological counseling	No
Mechanical bowel preparation	No	2–3 days after surgery
Preoperative fasting	Fasting of formula or milk for ≥6 h and 10% glucose water for ≥2 h before surgery	Fasting overnight before surgery
Reduce preoperative "separation anxiety"	Arrive at the operating room exchange area half an hour in advance, play on the playground in the exchange area, and be sedated before entering the operating room	No
Insert gastric tube and urinary tube	Post-anesthesia	Post-anesthesia
Temperature management	Adjust the operating room temperature half an hour before the children enter the operating room; The operating bed is heated with a heating blanket; Nasopharyngeal temperature was monitored throughout the process; The intraoperative flushing solution and intravenous infusion were heated	Control operating room temperature; The operating bed is heated with a heating blanket; Intraoperative flush solution and intravenous infusion were normal temperature
Intravenous fluid management	Goal-oriented liquid management	Administer fluids according to experience
Mode of anesthesia	General anesthesia combined with regional nerve block	General anesthesia
Anesthesia depth monitoring	Yes	No
Analgesic management	Postoperative analgesia pump, oral ibuprofen if necessary	No
Extubation of urinary tube	The first day after surgery	2–3 days after surgery
Extubation of gastric tube	The first day after surgery	Gastrointestinal decompression is reduced, can defecate and exhaust gas, generally in the postoperative 2–3 days
Extubation of abdominal drainage tube	2–3 days after surgery	3–5 days after surgery
Get out of bed early	The first day after surgery	generally about 3 days after surgery
Early eating	Lollipop was given 6 hours after surgery. Take formula milk on the first day after surgery	3 days after surgery

#### 2.2.3. Surgical methods

All operations were performed by the same surgical and anesthesia teams. Laparoscopic-assisted radical resection and hepatodochal jejunostomy were conducted in both groups, and the specific procedures were reported in previous studies (3).

### 2.3. Observation indicators

Intraoperative data included blood loss, operation time, conversion rate to laparotomy, and opioid dosage. Relevant postoperative data included FLACC pain assessment scale results on the first, second, and third days after surgery, time of gastric tube, urinary catheter, and abdominal drainage tube removal, time to first defecation after surgery, time to first eating after surgery, time to reach full food intake, CRP and albumin level (ALB), alanine aminotransferase (ALT) on the third and seventh postoperative day, postoperative complication rate, postoperative hospital stay, total treatment cost, and 30-day readmission rate.

### 2.4. Discharge criteria

The children were discharged when they were in good condition, could move freely out of bed, met physiological requirements through oral eating, had no drainage tubes, and their incisions had healed well. Blood routine examination results were normal, and biochemical indicators showed that liver function and bilirubin were normal or significantly better than before.

### 2.5. Follow-up

Outpatient and telephone follow-up were conducted to investigate the patient's feeding and defecation conditions, fever, abdominal pain, and other discomfort. The follow-up time was one month.

### 2.6. Statistical analysis

SPSS 21.0 statistical software was used for analysis. Normally distributed measurement data were expressed as *x* ± *s*, and pairwise comparison was conducted using the independent sample *t*-test. Non-normally distributed data were compared using the median and quartile, and comparison between two independent samples was conducted using the Mann–Whitney *U*-test. Fisher exact probability method was used to compare count data. *P* < 0.05 was considered statistically significant.

## 3. Results

### 3.1. General information of children

There were no significant differences in gender, age, weight, cyst diameter, and other general data between the two groups (Table 2).

**Table 2:** Comparison of preoperative general data between the two groups.

Group	Gender (male/female)	Age (years, mean ± SD)^a^	Body weight (kg, mean ± SD)	Cyst diameter (cm, mean ± SD)	Cases of inflammation of liver, biliary and pancreatic system (none/yes)^b^
ERAS group	3/12	3.4 ± 1.6	16.1 ± 4.0	2.8 ± 1.3	3/12
Traditional group	4/11	3.2 ± 1.5	14.9 ± 6.0	2.9 ± 1.0	2/13
*T*-value	–	28.0	24.4	25.5	–
*P*-value	1.000^c^	0.68	0.52	0.95	1.000^c^

^a^ All the patients in this study ranged in age from 3 months to 7 years.

^b^ Inflammation of the liver, biliary, and pancreatic systems: fever (excluding other systemic factors), abdominal pain, accompanied by elevated white blood cells and CRP indicated by blood routine or elevated ALT, total bilirubin, direct bilirubin, or amylase indicated by biochemistry.

^c^ Use Fisher exact probability method.

### 3.2. Changes of CRP, ALB and ALT levels in perioperative period

There were no significant differences in preoperative CRP, ALB, and ALT between the two groups. However, there were statistically significant differences in CRP, ALB, and ALT levels of the ERAS group on the 3rd and 7th day after surgery compared to the traditional group (Table 3).

**Table 3:** Comparison of perioperative CRP, ALB and ALT between the two groups.

Group	CRP (mg/L)			ALB (g/L)			ALT (g/L)		
	Perioperative	The 3rd day after surgery	The 7th day after surgery	Perioperative	The 3rd day after surgery	The 7th day after surgery	Perioperative	The 3rd day after surgery	The 7th day after surgery
ERAS group	22.9 (17.5, 42.1)	44.8 (39.8, 65.8)	13.9 ± 11.8	41.1 (39.8, 43.2)	35.5 ± 2.6	39.0 ± 1.9	30.4 (22.8, 45.3)	136.9 (105.7, 278.5)	37.2 ± 25.1
Traditional group	28.4 (19.1, 35.4)	72.4 (61.3, 108.0)	32.4 ± 19.2	40.6 (39.4, 41.8)	29.8 ± 4.5	34.1 ± 2.7	31.5 (23.6, 52.7)	265.8 (219.8, 321.4)	64.9 ± 30.0
T/U-value	104.0	44.0	30.2	82.0	22.5	32.7	102.5	44.0	34.5
*P*-value	0.744	0.004	0.001	0.217	<0.001	<0.001	0.683	0.004	0.004

### 3.3. Intraoperative conditions

There were statistically significant differences in opioid dosage between the two groups. However, there were no significant differences in intraoperative blood loss, operation time, and the number of patients transferred to open surgery (Table 4).

**Table 4:** Comparison of the dosage of opioids, intraoperative blood loss, operation time and the number of cases of conversion to laparotomy between the two groups.

Group	The dosage of opioids (μg)^a^	Intraoperative blood loss (ml)	Operation time (min)	The number of cases of conversion to laparotomy
ERAS group	2799.8 ± 860.1	12.7 ± 5.0	186.8 ± 35.2	0
Traditional group	3734.8 ± 799.0	11.5 ± 5.2	178.2 ± 47.1	0
*T*-value	27.8	28.0	25.9	–
*P*-value	0.005	0.50	0.576	–

^a^ The opioids in this study were all remifentanil.

### 3.4. Comparison of postoperative efficacy

The results of the FLACC pain assessment scale on the 1st and 2nd days after surgery, the extraction time of the gastric tube, catheter tube, and peritoneal drainage tube, the first postoperative defecation time, the first postoperative feeding time, full feeding time, postoperative hospitalization time, and total treatment cost were significantly different between the ERAS group and the traditional group. However, there was no statistically significant difference in the results of the FLACC pain assessment scale on the 3rd day, the incidence of postoperative complications, or the readmission rate within 30 days after surgery (Table 5).

**Table 5:** Comparison of postoperative efficacy.

Group	The results of the FLACC scale on the 1st day^a^	The results of the FLACC scale on the 2nd day^a^	The results of the FLACC scale on the 3rd day^a^	The time of gastric tube removal (h)	The time of urinary catheter removal (h)	The time of abdominal drainage tube removal (h)	The time of first defecation after operation (h)
ERAS group	3.0 (2.0, 3.0)	1.0 (1.0, 2.0)	1.0 (1.0, 1.0)	20.8 ± 2.2	45.0 (43.0, 49.0)	63.4 ± 9.2	32.0 (29.0, 33.0)
Traditional group	4.0 (3.0, 5.0)	3.0 (2.0, 3.0)	1.0 (1.0, 1.0)	67.4 ± 4.9	80.0 (52.0, 90.0)	95.4 ± 11.8	61.0 (35.0, 68.0)
*T*/*U*-value	36.5	21.0	100.5	25.4	16.5	33.8	40.0
*P*-value	0.001	<0.001	0.624	<0.001	<0.001	<0.001	0.002

**Table:** 

Group	The time of first eating after operation (h)	The time to reach full food intake (d)	The incidence of postoperative complications (Yes/No)^b^	Postoperative hospital stay (d)	Total treatment cost (thousand RMB)	The rate of readmission within 30 days
ERAS group	28.0 (26.0, 32.0)	6.0 (5.0, 7.0)	1/14	7.2 ± 1.3	37.6 ± 5.7	0
Traditional group	53.0 (48.0, 68.0)	8.0 (6.0, 9.0)	1/14	9.4 ± 2.0	47.4 ± 5.4	0
*T*/*U*-value	13.0	52.5	–	31.1	27.9	–
*P*-value	<0.001	0.01	1.000^c^	<0.001	<0.001	–

^a^FLACC pain assessment scale was evaluated independently by two fixed medical staff at 8:00, 12:00 and 15:00 each day, and the results were averaged and included by rounding method.

^b^ Postoperative complications: intestinal leakage, biliary leakage, pancreatic leakage, intestinal obstruction.

^c^ Use Fisher exact probability method.

## 4. Discussion

ERAS is a comprehensive approach that covers preoperative, intraoperative, and postoperative aspects (4). It involves detailed preoperative education, strengthened anesthesia management and intraoperative treatment measures, and optimized postoperative management.

### 4.1. Preoperative education

Preoperative anxiety is characterized by subjective feelings of nervousness, irritability, sadness, and unhappiness. Studies have shown that preoperative anxiety negatively impacts the psychology and physiology of children, with 60%–80% of children undergoing anesthesia and surgery experiencing it. Preoperative anxiety is associated with adverse behavior changes, such as nightmares, separation anxiety, eating disorders, increased fear of doctors, and other negative consequences (5). The degree of preoperative anxiety in pediatric patients is particularly high, with young age, previous surgical history, anesthesia, ambulatory surgical environment, and parental anxiety as independent risk factors for it (6). Anxiety in children usually stem from the unfamiliar environment rather than the severity of the disease, while parents' anxiety is often due to the disease itself and the fear of surgery. Good preoperative education can provide relevant knowledge about CC, improve children's and their parents' confidence in the treatment, may reduce fear and tension regarding the disease treatment, increase children's and their families' cooperation in the treatment, and facilitate smooth operation and related treatment.

### 4.2. Mechanical bowel preparation (MBP)

MBP refers to various forms of enemas or laxative administration to remove stool and gas contents in the intestine. The clean reflux enema is the most commonly used MBP method in children. However, this operation can cause pain, discomfort, and increased anxiety in children. Almost all children or parents describe preoperative MBP as the most unpleasant part of hospitalization (7). Excessive gas inhalation during crying can also lead to flatulence, which can affect the surgical visual field. Some children may have lavage liquid remaining in the transverse colon, leading to transverse colon dilation, interference with the surgical visual field, and affecting the operation. A prospective randomized study on whether to perform MBP before surgery for adult gastric cancer found that MBP not only changes the intestinal flora composition of patients but also increases the incidence of postoperative delirium (8). In colorectal surgery in children, the absence of MBP before surgery does not increase the risk of anastomotic fistula (9). The absence of MBP in children undergoing cystoplasty with sigmoid, ileocecal or ileum does not increase the risk of infection or anastomotic complications (10). In this study, the ERAS group did not receive MBP, and there was no statistically significant difference between the two groups in operation time, intraoperative blood loss, incidence of postoperative complications, and readmission rate within 30 days, confirming the safety of avoiding MBP before surgery.

### 4.3. Fasting time before surgery

The digestive system function of infants and young children is not mature, and their gastrointestinal peristalsis is faster than that of adults. However, appropriate shortening of the preoperative fasting time will not increase the risk of aspiration. Compared with children who were deprived of liquids for more than 6 h, the consumption of clear liquids did not increase stomach volume or potential of hydrogen value until 2 h before surgery (11). The intake of carbohydrate drinks before elective surgery has been shown to reduce the incidence of insulin resistance, nausea, and vomiting after surgery, maintain glycogen reserves, and reduce protein breakdown (12). A prospective study of patients undergoing thyroidectomy found that, compared with patients who received carbohydrate beverages 2 h before surgery, intraoperative blood glucose levels were significantly higher in patients who did not receive carbohydrate beverages, as well as the incidence and severity of postoperative nausea and vomiting and length of intensive care unit stay (13). Most of the children who fasted for a long time showed obvious thirst, hunger, and irritability, which led to some children secretly eating and the cancellation of surgery. In this study, compared with the traditional group, the ERAS group did not find an increase in the risk of vomiting and aspiration during anesthesia and reduced the amount and time of infusion the night before surgery. This allowed children and their parents to get enough rest and facilitate postoperative rehabilitation and nursing. It can also reduce the occurrence of accidents such as the shedding of infusion needles and soft tissue swelling at the infusion site caused by crying and struggling with hunger. In this study, if the patient was not first in line on the day of surgery, the ERAS multidisciplinary team fully utilized the advantages of teamwork. After communicating with the anesthesia department, the medical staff of ERAS gave the child glucose water and minimized the fasting time before surgery according to the schedule of the operating room, truly embodying the concept of ERAS.

### 4.4. Intraoperative management

Throughout the treatment of CC, the most intense stress response is observed during the surgical procedure. Proper depth of anesthesia can decrease the stress response, decrease cerebral oxygen metabolism, and prevent drastic hemodynamic fluctuations, highlighting the significance of anesthesia management. In the traditional group, the dosage of anesthetic drugs was adjusted based on the anesthesiologist's experience, as well as heart rate and circulation conditions, and feedback from the surgeon regarding the degree of muscle relaxation in children to adjust the depth of anesthesia. The ERAS group, on the other hand, maintains appropriate anesthesia depth through the use of an anesthesia depth monitor, with bispectral index being the most commonly employed method, and has a positive clinical significance in reducing the incidence of cognitive dysfunction during and after surgery (14). The dose of narcotic drugs was adjusted using bispectral index results, and general anesthesia combined with regional nerve block significantly decreased the intraoperative dose of opioid drugs. Due to the inhibitory effect of exogenous opioids on intestinal opioid receptors, intestinal peristalsis can be inhibited (15). Multimodal analgesia for pain, especially with regional nerve block, significantly reduced the dosage and side effects of opioids and enhanced the recovery time of intestinal function (16). The dose of opioids used in the ERAS group in this study was significantly lower than that in the traditional group, which decreased the incidence of postoperative nausea, vomiting, and inhibition of gastrointestinal peristalsis, and accelerated postoperative recovery of gastrointestinal function, thereby corroborating this finding.

### 4.5. Postoperative management

Patients in the ERAS group who had urethral catheter removal and bladder massage on the first day after surgery did not show any urinary retention or re-indwelling urethral catheter, indicating the feasibility of early urethral catheter removal. A study on 3D laparoscopic excision for choledochal cysts found that early catheter removal was safe and feasible (17). Early removal of the urinary tube can reduce the pain and discomfort caused by the tube, making children more willing to participate in early postoperative activities.

Traditionally, gastric tubes are placed after surgery to relieve gastrointestinal obstruction and reduce gastrointestinal burden, accelerating the recovery of gastrointestinal function. However, gastric tubes may lead to throat pain and discomfort, and phlegm is difficult to cough up. Prolonged indenturement of the gastric tube can delay eating time and is not conducive to the early recovery of intestinal function (18). Chewing gum after surgery can significantly shorten the time of first postoperative bowel movement and length of hospital stay in gynecological cancer patients (19). Early postoperative feeding does not increase the risk of abdominal distension, vomiting, or anastomotic fistula, and can promote the recovery of gastrointestinal function (20). In this study, the ERAS group showed significant improvement compared to the traditional group in terms of the time of first bowel movement after surgery, the time of first postoperative feeding, and the time of reaching full feeding without increasing complications, confirming the feasibility of early gastric tube extraction and early feeding.

Placement of an abdominal drainage tube will inevitably increase pain in children. Fear and pain can prolong bed rest time after surgery, increase the chance of lung infection, and affect the recovery of intestinal function. Long-term indwelling tubes are also prone to cause abdominal infection. It has been reported that in special cases such as biliary tract system infection, hepatic duct angioplasty, and deep embedding of the cyst distal end into the pancreas, there is no need to place a drainage tube (21). The next step of this study is to explore the feasibility of not placing an abdominal drainage tube to further implement the concept of rapid rehabilitation.

Postoperative pain increases the stress response of children, making them afraid to turn over and walk, interfering with their sleep, and not conducive to rapid postoperative recovery. Therefore, postoperative analgesia is an important link to achieving rapid recovery. However, young children do not accurately express pain and its extent, and in most cases respond by crying. To objectively assess the degree of pain in children, we adopted the FLACC pain assessment scale with the cooperation of family members, excluding children's hunger, thirst, and anxiety. The FLACC pain assessment scale has shown sufficient feasibility and clinical practicability in evaluating pain in the pediatric intensive care unit (22), and is a valuable method for quantifying pain after adenoid tonsillectomy in children (23). Through the scale results, we found that the scores of the 1st and 2nd day after surgery in the ERAS group were lower than those in the traditional group, indicating that the postoperative analgesic patterns in the ERAS group had positive clinical significance. There was no statistically significant difference between the two groups in the score on the 3rd day after surgery, which may be consistent with the gradual improvement of the condition and relief of pain in the children after surgery, reflecting the need to pay attention to analgesia in the first 2 days after surgery. A study investigating the impact of auditory distraction methods on postoperative pain and anxiety in children found that classical music, Turkish music, and audiobook methods were effective in alleviating postoperative pain and anxiety in children. Among these methods, listening to classical music was found to be the most effective in relieving postoperative pain in children (24). The next step in this study is to further improve multimodal analgesia, with the aim of minimizing drug use while ensuring clinical analgesia, in order to achieve rapid recovery.

However, there are some limitations in this study, including its retrospective, single-center design and small sample size, as well as a relatively short follow-up time. To address these limitations, we plan to conduct a prospective, multicenter study with a larger sample size and longer follow-up period to ensure more accurate results and robust statistical analysis.

## 5. Conclusion

The results of this study indicate that the application of ERAS in laparoscopic-assisted radical resection of type I CC not only eliminates the need for MBP but also reduces pain in children. Compared to the traditional group, the ERAS group showed advantages in the results of the FLACC pain assessment scale on the 1st and 2nd postoperative days, time to first eating after surgery, time to reach full food intake, time of first defecation after operation, length of postoperative hospital stay, and total cost of treatment, without an increase in the incidence of complications after surgery. These findings suggest that the ERAS concept is an effective and safe approach for laparoscopic-assisted radical resection of type I CC.

## Data Availability

The raw data supporting the conclusions of this article will be made available by the authors, without undue reservation.
